# Spatial Reorganization of the Endoplasmic Reticulum during Mitosis Relies on Mitotic Kinase Cyclin A in the Early *Drosophila* Embryo

**DOI:** 10.1371/journal.pone.0117859

**Published:** 2015-02-17

**Authors:** Zane J. Bergman, Justin D. Mclaurin, Anthony S. Eritano, Brittany M. Johnson, Amanda Q. Sims, Blake Riggs

**Affiliations:** Department of Biology, San Francisco State University, 1600 Holloway Ave., San Francisco, California, 94132, United States of America; University of Toronto, CANADA

## Abstract

Mitotic cyclin-dependent kinase with their cyclin partners (cyclin:Cdks) are the master regulators of cell cycle progression responsible for regulating a host of activities during mitosis. Nuclear mitotic events, including chromosome condensation and segregation have been directly linked to Cdk activity. However, the regulation and timing of cytoplasmic mitotic events by cyclin:Cdks is poorly understood. In order to examine these mitotic cytoplasmic events, we looked at the dramatic changes in the endoplasmic reticulum (ER) during mitosis in the early *Drosophila* embryo. The dynamic changes of the ER can be arrested in an interphase state by inhibition of either DNA or protein synthesis. Here we show that this block can be alleviated by micro-injection of Cyclin A (CycA) in which defined mitotic ER clusters gathered at the spindle poles. Conversely, micro-injection of Cyclin B (CycB) did not affect spatial reorganization of the ER, suggesting CycA possesses the ability to initiate mitotic ER events in the cytoplasm. Additionally, RNAi-mediated simultaneous inhibition of all 3 mitotic cyclins (A, B and B3) blocked spatial reorganization of the ER. Our results suggest that mitotic ER reorganization events rely on CycA and that control and timing of nuclear and cytoplasmic events during mitosis may be defined by release of CycA from the nucleus as a consequence of breakdown of the nuclear envelope.

## Introduction

Entry into mitosis displays a dramatic remodeling of cellular structure and organization. These activities include nuclear events such as chromosome condensation and nuclear envelope breakdown (NEB) and cytoplasmic events including spindle assembly, Golgi apparatus breakdown, and changes in Endoplasmic Reticulum (ER) structure and localization [1]. Essential in this process is the timing and stepwise coordination of both nuclear and cytoplasmic events for proper cell division and distribution of their contents to the newly formed daughter cells. Over the last decade, there have been several studies focused on the regulators of these activities during mitosis [for reviews see 2–4], however the coordination of nuclear and cytoplasmic events at defined stages during mitosis is poorly understood.

Regulation of cell cycle events is controlled by cyclins and their associated cyclin-dependent kinases (cyclin:Cdk). Oscillation of cyclin:Cdk activity drives progression through the cell cycle and Cdk1 activity has been implicated in the coupling of nuclear and cytoplasmic events during mitosis [5–7]. Studies involving cytoplasmic organelle targets including changes of the ER and Golgi apparatus have suggested that mitotic cyclin:Cdk complexes may control the dramatic events seen during mitosis [8]. An *in vitro* examination of ER network formation showed that membrane isolated from mitotic *Xenopus* egg extracts containing a non-degradable Cyclin B (CycB1Δ90) mutant protein are unable to properly organize into an ER network [9]. A recent study also outlined that reformation of the Golgi apparatus and ER at telophase requires the inactivation of Cdk1 by the inhibitory kinase Myt1 [10]. Cyclin:Cdk1 has also been implicated in nuclear envelope breakdown and reformation [11,12]. Most importantly, a mitotic regulator or particular mitotic cyclin *in vivo* that drives the changes in localization and structure of these organelles during mitosis has not been identified.

The ER is a highly dynamic organelle, and displays dramatic changes in both its structure and localization during mitosis. In interphase, the ER exists as an interconnected network of sheet-like cisternae and an array of tubules that are continuous with the nuclear envelope and stretches to the periphery of the cell [13,14]. Upon entry into mitosis, at the point of NEB, the ER undergoes a dramatic structural transformation which includes a change from sheet-like cisternae connected by tubules to an extended cisternae with a reduction in tubule structures [15]. In addition to this morphological transformation at the nucleus, the ER also experiences a spatial reorganization of its cytoplasmic localization as mitosis progresses. The ER accumulates and aligns along the mitotic spindle early in mitosis and, in some organisms, along the central spindle/midbody later in mitosis [16,17]. Recent studies have shown that the targeting of ER tubules to the chromatin during late mitosis is important for nuclear envelope reformation and organization of nuclear pore complex proteins [18–20]. The dynamic changes experienced by the ER as well as other cytoplasmic structures during the cell cycle have been well documented, however it is still unclear how these changes are regulated.

Here we provide key insight into the regulation of ER spatial reorganization and morphology changes during mitosis utilizing the early syncytial blastoderm stage of *Drosophila* embryos. Early *Drosophila* development occurs in a syncytium beginning with 13 synchronous, rapid nuclear divisions ending with each nucleus encapsulated into a cell at the 14^th^ division. After nine divisions in the interior of the embryo, nuclear divisions 10–13 occur at the cortex just beneath the plasma membrane [21]. In these syncytial divisions of the early *Drosophila* embryo, the nuclei essentially oscillate between S-phase and M-phase with no clearly discernible gap phases [22]. During these cortical divisions, ER membranes and other components of the secretory membrane system are equally allocated to daughter nuclei and display a dramatic change in morphology during mitosis similar to what is seen in other systems [16,23]. In this study, we demonstrate that ER dynamics are in frame and coordinated with the cell cycle. Inhibition of all three mitotic cyclins (A, B, and B3) by injection of double-stranded RNA arrests the embryo in interphase, and blocks ER spatial reorganization activity. In addition, we show that Cyclin A, not Cyclin B, is sufficient to drive early mitotic ER spatial reorganization events in the presence of an interphase arrest. Taken together, we show a cytoplasmic target of Cyclin A:Cdk activity involving mitotic ER spatial reorganization at the spindle poles during prometaphase.

## Results

### Mitotic ER spatial reorganization along the spindle and at poles coincides with NEB

Several studies have highlighted the dynamic changes experienced by the ER during the cell cycle [15,24,25]. Yet to be identified is the link between cell cycle regulatory networks and the stepwise changes in ER structure and positioning during mitosis. In order to begin characterizing a pathway responsible for mitotic cytoplasmic events, we utilized the cortical divisions of the early *Drosophila* embryo to observe nuclear and cytoplasmic events during several rounds of mitosis. The *Drosophila* syncytial blastoderm is well established for the study of mitosis and has led to several discoveries surrounding mitotic events that correlate with vertebrate systems [26–28]. We used time-lapse confocal microscopy and imaged the distinct morphologies of the ER during the cortical syncytial embryonic stages. The behavior of the ER during these syncytial divisions was originally described by Bobinnec and colleagues [16], wherein they followed the ER-lumenal protein Protein Disulfide Isomerase (Pdi) fused to GFP as the embryo cycled through these mitoses. In this study, we crossed the Pdi-GFP line with a line expressing a DNA marker, His2Av-RFP (H2-RFP) to monitor cytoplasmic and nuclear events, respectively.

**Fig 1:**
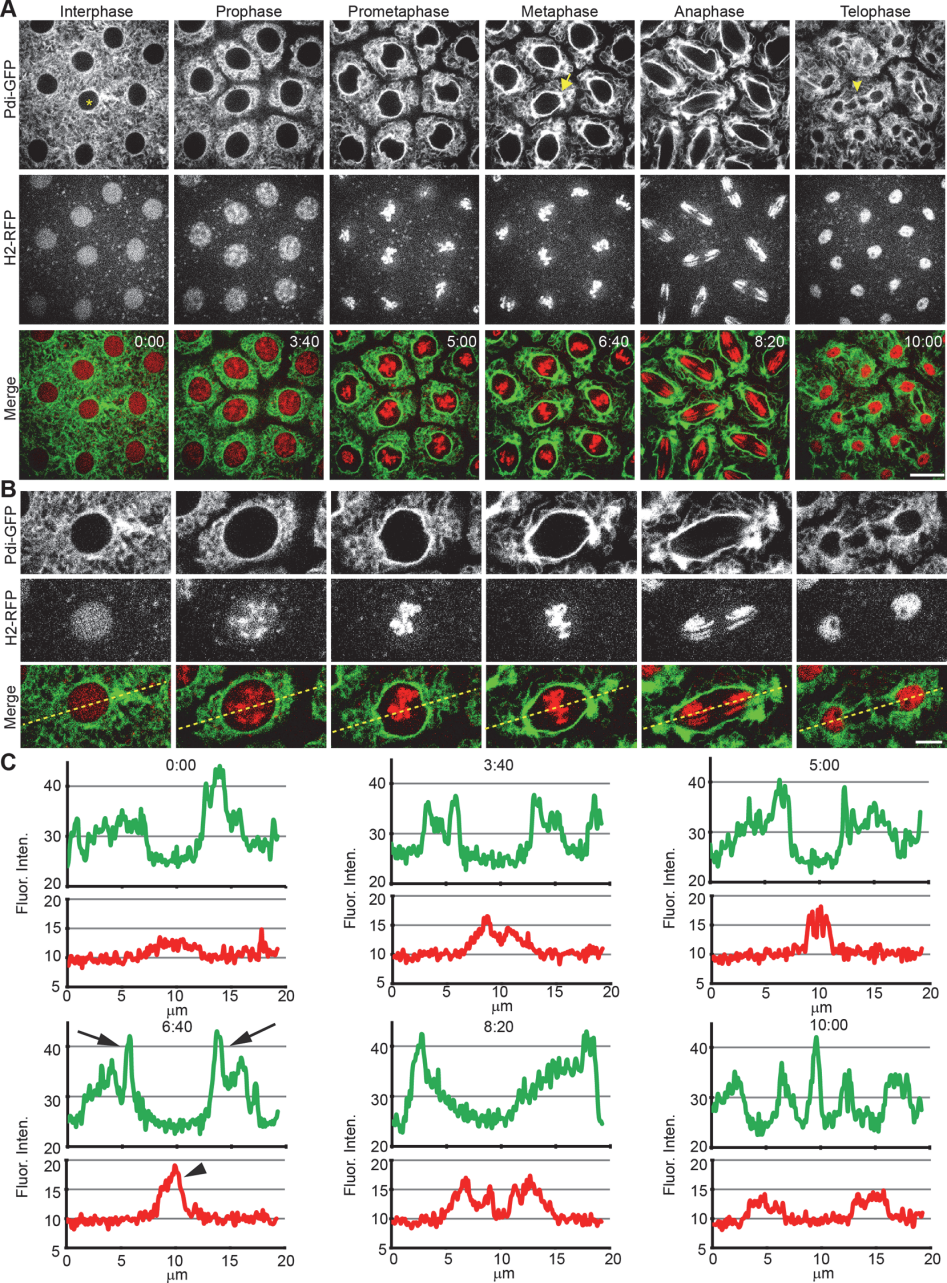
The ER displays dramatic structural and localization changes during mitosis in the early *Drosophila* embryo. (**A**) Mitotic ER dynamics were examined in cycle 11 transgenic *Drosophila* embryos expressing the ER marker Pdi-GFP and the DNA marker H2-RFP using time-lapse confocal microscopy. Phases of mitosis are listed at the top with relative time (min:sec) listed in the merge panels. During interphase, ER (green) was initially spread loosely around the nucleus. Upon entry into mitosis, ER accumulates around the nucleus and was rapidly converted to thick, perinuclear cisternae upon chromosome condensation (red) and prophase onset. in prometaphase, the ER membrane reorganizes with the developing mitotic spindle and begins to accumulate at the spindle poles. At metaphase and anaphase the ER is aligned with the mitotic spindle and displays movement towards the spindle poles (arrow). During late anaphase and telophase, the ER sees a rapid localization around the segregated, decondensing chromosomes and a localization at the central spindle / midbody (arrowhead). Scale bar is 10 μm. (**B**) High magnification of mitotic ER changes following a single nucleus used for quantification of ER movements shown in C (asterisk in A). Yellow line denotes fluorescence trace shown in C. Scale bar is 5 μm. (**C**) Fluorescence intensity trace of ER (green line) and chromosomes (red line) along 20 μm of the developing embryo. ER fluorescence is maximal just adjacent to the nuclear space, but is excluded from the nucleus. During interphase, the ER is evenly distributed throughout the cytoplasm. Intensity around the nucleus increases during mitosis and follows the extension of the spindle. Pdi-GFP signal intensity reached maximum during metaphase (arrows). Condensation and alignment of chromosomes at the metaphase plate are marked by the arrowhead. At telophase, two new nuclear envelopes are formed with a large peak at the remaining midbody.

Our observations of the ER during the cortical syncytial divisions align well with previous work and we can more precisely denote nuclear mitotic events with the addition of H2-RFP (Fig. 1). A field of nuclei in cycle 11 displayed the ER changing from a large mesh-like network distributed uniformly during interphase, which gathered close to the nuclear envelope as the embryo entered mitosis (Fig. 1A, S1 Movie). At prometaphase, there are marked changes in both position and structure of the ER. At the region adjacent to the nucleus, the ER became increasingly thick around the perispindle and a large amount of ER concentrated at each pole of the spindle during metaphase (arrow). When the spindle elongated during anaphase, the ER at the perispindle region followed closely with the segregating chromosomes. More distal ER was not directly tied to spindle movement. Upon telophase, daughter nuclei were surrounded by ER with a bright staining of ER at the spindle mid-zone (arrowhead). The ER then spread into a less defined meshwork and started the next interphase. Fig. 1B shows a high magnification inset of a single nucleus (Fig. 1A, asterisk) at cycle 11 and relative time from the end of the previous mitosis.

In order to follow ER reorganization events during the cell cycle, we quantified these morphological changes observed by measuring the raw fluorescence intensity of both Pdi-GFP and H2-RFP in a one-pixel wide line, 20μm in length through the nucleus (Fig. 1B, dashed yellow line). Quantification of H2-RFP signal (red) was used as a marker for cell cycle progression, with a small peak at 10 μm representing the diffuse H2 signal in the nucleus during interphase. As the embryo entered mitosis, there was a marked increase in the H2 signal peak at 10 μm as the chromosomes condensed and aligned at the metaphase plate (Fig. 1C, time point 6:40, arrowhead). There was a gradual widening of the signal peak towards 5 μm and 15 μm as the chromosomes segregated towards opposite poles during anaphase and the signal decreased as the chromosomes decondensed at telophase. The intensity of Pdi-GFP (green) around the nucleus increased steadily from prophase and peaked in metaphase at 5 μm and 15 μm corresponding to the location of the spindle poles. (Fig. 1C, time point 6:40, arrows). These GFP peaks then separated, along the axis as the nucleus proceeded through anaphase. At telophase, two new nuclear envelopes were observed forming around the diffusing RFP signal, with a bright spot at the spindle mid-zone. This pattern of fluorescence is typical of cycle 11 nuclei and was observed at the other cortical divisions, albeit at different scales.

**Fig 2:**
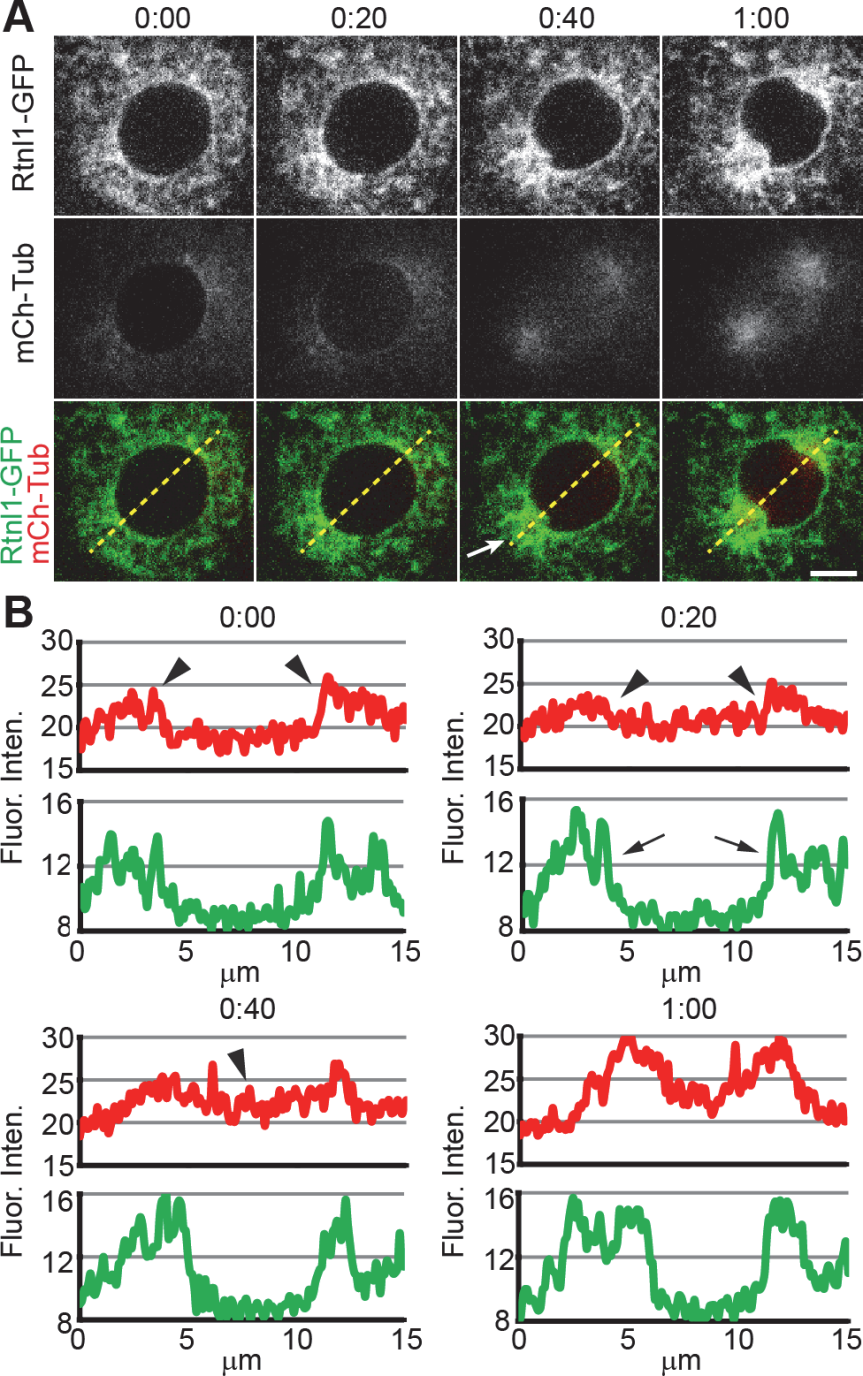
Mitotic ER rearrangements do not occur until after NEB. (**A**) An embryo expressing Rtnl1-GFP and mCh-Tub was observed during cycle 11. Tubulin entered the nuclear space at the 20 second time-point, signaling nuclear envelope breakdown prior to accumulation of ER at the centrosome. At the 40 second time point ER began its rearrangement at the centrosome (arrow). Yellow dotted line denotes fluorescence trace shown in B. Scale bar is 5 μm. (**B**) Fluorescence intensity traces of Rtnl1 (green line) and mCh-Tub (red line). mCh-Tub displays an intensity peak at 5 μm and 10 μm before NEB (arrowheads). At 20 seconds, mCh-Tub intensity becomes flat indicating NEB, while Rtnl1 intensity begins to form peaks at 5 μm and 10 μm (arrows). Rtnl1 intensity continues to rise and mCh-Tub intensity also rises between 5 μm and 10 μm indicating mitotic spindle formation. Time is in min:sec.

We closely examined the coordination of mitotic ER changes with nuclear envelope breakdown (NEB) during the early phases of mitosis. Here we used a protein trap line of the ER shaping protein, Reticulon-like 1 (Rtnl1-GFP) [29] as a marker for ER membrane movement. This line was created in a screen that fused a GFP tag in frame with the endogenous Rtnl1, which is embedded in the cytoplasmic face of the ER and co-localizes with ER structures [30]. We crossed this line to a line containing a UAS-mCherry-tubulin (mCh-Tub). The expression of the mCh-Tub was driven in the embryo by crossing this line to a maternal triple-driver line (see Methods). We examined F1 embryos from this line to follow the specific timing of cytoplasmic events (Fig. 2A). A prior study has demonstrated that using entry of injected Rhodamine-labeled tubulin into the nuclear envelope space is an accurate measurement of timing of NEB in the early *Drosophila* embryo [31]. We found that the dramatic ER changes seen at mitosis quickly followed NEB. At prophase, labeled tubulin is excluded from the nuclear space and found solely in the cytoplasm indicative of an intact nuclear envelope (Fig. 2A, time point 0:00). At this stage, there had been no reorganization of the ER network or accumulation at the poles. However, 20 seconds later, labeled tubulin began to rush into the nuclear interior indicating the start of NEB (Fig. 2A). At time point 0:40, centrosomes and asters aligned at the poles and the mitotic spindle had begun to form. In addition, ER membrane had begun to accumulate at the centrosomes (Fig. 2A, arrow). We also quantified the movement of ER with respect to NEB as shown in Fig. 2B. Measurement of labeled tubulin (red line) displayed a slight decrease in intensity between 5 μm and 10 μm corresponding to an intact nuclear envelope (Fig. 2B, time point 0:00, arrowheads). At the 20 second time point the fluorescence intensity flattened out indicating that mCh-Tub had entered the nuclear space signaling NEB (Fig. 2B, arrowheads). At this time point, measurement of Rtnl1 fluorescent intensity (green line) displayed minor peaks starting to form at 5 μm and 10 μm indicative of localization at the spindle poles (arrows). Rtnl1 peak intensity at 5 μm and 10 μm increased at the later time points, 0:40 seconds and 1:00 minute, while tubulin intensity (red line) showed a increase from 5 μm through 10 μm indicative of mitotic spindle formation (Fig. 2B, time point 0:40, arrowhead). Taken together, these data suggest that ER spatial events and movement of the ER to the spindle poles are precisely timed during mitosis occurring concurrently with NEB.

### Extended ER Cisternae Form at the Poles During Mitosis

**Fig 3:**
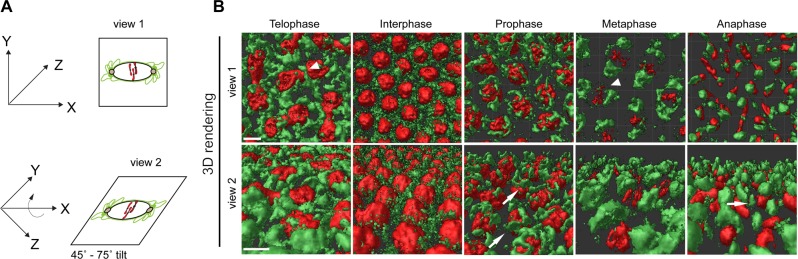
3D reconstruction of ER structural changes display a clustering of extended cisternae at the spindle poles during metaphase. Embryos expressing Pdi-GFP (green) and H2-RFP (red) were fixed and imaged using confocal microscopy. (**A**) Upper panels (view 1) represent a top view of the nucleus and surrounding ER along the xy-plane and bottom panels (view 2) show the nucleus and ER ~45° -75° tilt along the in the y-plane. Embryos were imaged in the z-direction with a step size of 0.1 μm and subject to 3D reconstruction software. (**B**) At telophase of cycle 11, the ER is globular and spread along the reforming nuclear envelope and at the midbody (view 1, arrowhead). Exiting mitosis, at interphase, the ER is spread loosely through the cytoplasm outline the nuclear envelope. At prophase, the ER becomes defined and begins to cluster and propagate apically at the spindle poles. These clusters are not uniform in size and appear to be sheet-like structures (view 2, arrows). At metaphase, the clusters are found at the spindle poles and appear to be connected along the spindle area forming a sheath (view 1, arrow). In anaphase, ER cisternal clusters appear with the segregating chromosomes and at the midbody (view 2, arrow). Scale bar is 5 μm.

Several studies have examined the structural morphological changes of the ER during mitosis, however the exact nature of these changes has been the source of much debate [15,25,32]. Earlier studies described mitotic ER as being highly branched and tubular [32]. However, a recent study described the organization of the ER during mitosis as consisting mainly of cisternal sheets and a lesser degree of tubules [15]. In our 2D confocal imaging, there appeared to be the presence of highly tubular structures emanating from the spindle poles during metaphase suggesting a greater tubular network than previously reported [15,16] (Fig. 1B, top row). In order to investigate the structural nature of these ER tubules at the poles, we examined the ER during the different stages of the cell cycle in Pdi-GFP / H2-RFP transgenic embryos. For this, embryos were fixed according to published protocols [27, 33] and imaged using laser-scanning confocal microscopy. 0.1 μm optical z-steps were imaged to a depth of 10 μm from the cortex. These images were then processed using 3D reconstruction software (see Methods) to examine the structure of the ER during mitosis. Fig. 3A (S2 Movie) displayed the orientation of our 3D rendering of the ER during mitosis with view 1 along the x and y plane (top row) and view 2 from a ~45° -75° tilt along the x-axis (bottom row). At telophase, the ER appeared very globular surrounding the decondensed chromosomes, and a tubular-like structure appeared at the site of the midbody (Fig. 3B, top row, arrowhead). As the embryo entered interphase, the ER displayed a loosely connected punctate structure surrounding each nucleus. During prophase, the ER appeared to form more defined tubular structures apical of the nucleus, however as the image was rotated, these tubules are not uniform in size and appeared wide in some areas which are more reminiscent of sheet-like structures (Fig. 3B, bottom row, arrows). At metaphase, the ER formed defined large clusters at the spindle poles connected along the edge of the spindle area (Fig. 3, top panel arrowhead). Rotation along the y-axis (Fig. 3B, bottom panel) showed that the ER formed large globular domains or clusters at the spindle poles and there was a general lack of tubule structures present. As chromsomes segregated during anaphase, the ER clusters remained associated with the chromosomes and the spindle pole. The ER clusters appeared to be apical of the segregating chromosomes and in line with the ER population at the midbody (Fig. 3B, bottom panel arrows). In comparing our 3D structural analysis of the clusters at the poles with the 2D tubular structures it appears that these globular domains are hollow and represent cisternal sheets folded in clusters attached to the poles via an unknown mechanism. This result is confirmed by EM imaging of the structural transitions of the ER performed by Bobbinec in which they describe the ER transitions as changes from a tubular organization to formation of large puffy structures [16].

### ER dynamics are arrested in the presence of cell cycle inhibitors

The dynamics displayed by the ER during mitosis must be timed and well-coordinated by a temporal mechanism. The likely candidates for this coordination are the cyclin-dependent kinases. Towards this idea, a prior study added a non-degradable cyclin B1Δ90 to *in vitro* Xenopus egg extracts and saw a change in ER dynamics [9,24]. In order to investigate the link between the cell cycle and ER dynamics *in vivo*, we micro-injected known cell cycle inhibitors to test their effects on ER organization in Pdi-GFP / H2-RFP embryos.

**Fig 4:**
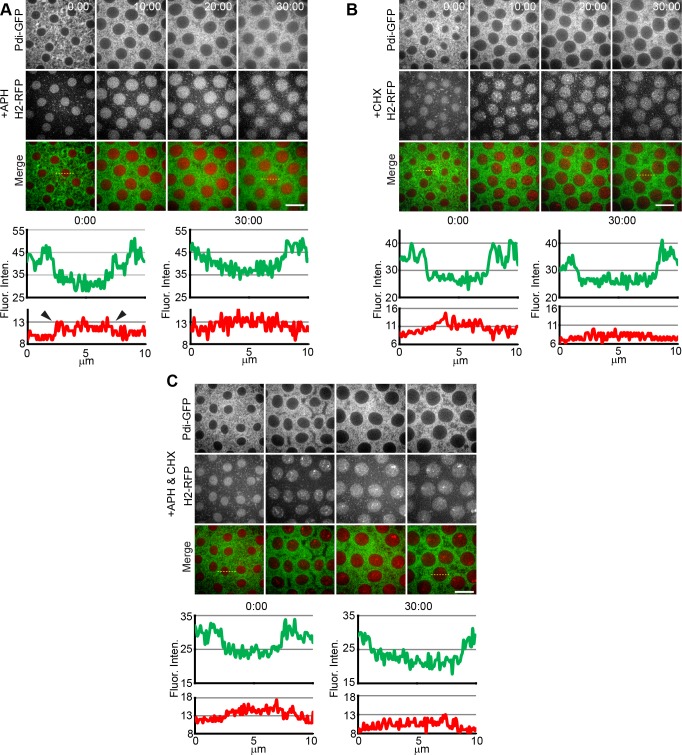
Arresting the *Drosophila* embryo in interphase maintains the ER in an interphase-like state. (**A**) Time-lapse confocal images of a Pdi-GFP (green) / H2-RFP (red) transgenic embryo injected at metaphase cycle 10 with the DNA replication inhibitor, aphidicolin (APH) and viewed during cycle 11. APH, arrests the embryo in S-phase of cycle 11. In the presence of APH, the ER displayed a loose uniform distribution around the nuclei denoting an interphase-like state. This interphase-like state of the ER persists for greater than 30 minutes without any changes to either localization or structure. This is quantified in the fluorescence intensity traces below (see yellow dotted-lines in merged images). H2-RFP signal inside the nucleus does not increase over this time period as well (arrowheads). (**B**) Time-lapse confocal images of a Pdi-GFP (green) / H2-RFP (red) embryo injected with the protein synthesis inhibitor cycloheximide (CHX) at metaphase of cycle 10 and viewed during interphase of the following cycle. Similar to APH, CHX induced arrest which maintained the ER in an interphase-like state. This is quantified below, as in A. (**C**) Similar background and approach as A and B. Embryos were injected with an APH+CHX cocktail. ER membrane maintained an interphase-like organization as seen in APH injections alone. Scale bar is 10 μm. Time is in min:sec.

Micro-injection of aphidicolin (APH), a DNA synthesis inhibitor, during mitosis of cycle 10 arrested embryos (8/9 embryos) in an interphase-like state in the subsequent cycle (Fig. 4A). This arrest lasted for the duration of observation (>30 min). During the arrest, the ER did not accumulate towards the nuclear envelope or rearrange at the centrosome, like in mitosis, nor did it gather adjacently to the spindle. Chromosomes did not condense, signaling a block prior to mitosis. This was quantified at the start of the arrest and at 30 minutes (Fig. 4A, lower panels, arrowheads). The H2-RFP measurements displayed a small peak at 5 μm, corresponding to a diffuse H2 signal in the nucleus, indicative of interphase. The nuclear envelope did not break down during this arrest either, as detected by GFP-Lamin (data not shown). Control injections showed no noticeable effects on timing of cytoplasmic or nuclear events or progression of the cell cycle in the early embryo (S2 Fig.). These results indicate that the syncytium must progress at least beyond S-phase to begin ER rearrangement.

The synthesis of a protein factor important for cell cycle progression could also be necessary for the timing pathway of mitotic ER dynamics. Micro-injection of cycloheximide (CHX), a translation inhibitor, during mitosis of cycle 10 arrested embryos during cycle 11 (9/9 embryos). A similar interphase-like arrest was seen, where the ER did not undergo any changes and the DNA stayed mainly uncondensed (Fig. 4B). Injection of both APH and CHX (7/7 embryos) similarly arrested the embryo in an interphase-like state (Fig. 4C, S3 Movie) with the nuclear envelope intact (S1 Fig.). This gives evidence that a protein or proteins must be synthesized in order for the syncytium to advance both nuclear and cytoplasmic events towards mitosis. Cyclins are constantly degraded and translated during the syncytial divisions [34] whereas other mitotic kinases, such as Polo-like kinase, levels are relatively constant [35], making cyclins more likely candidates for the regulatory factors controlling ER dynamics.

**Fig 5:**
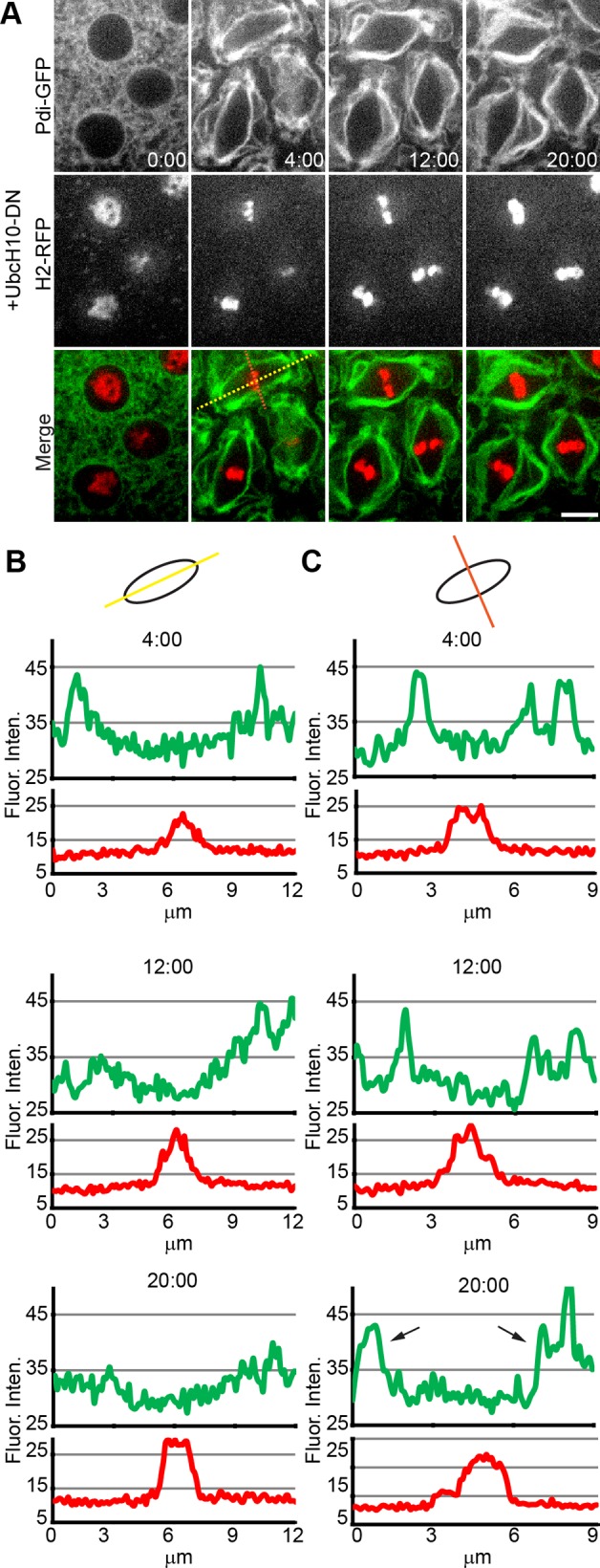
Inhibition of the APC/C maintains the ER in a mitotic state. (**A**) Time-lapse confocal images of a cycle 10 Pdi-GFP / H2-RFP transgenic embryo following micro-injection of a dominant-negative form of UbcH10 just prior to entry into mitosis, eventually arresting the embryo in metaphase. The ER displayed normal structural organization and localization changes early in mitosis and relocated to the mitotic spindle upon nuclear envelope breakdown. The embryo then arrested at metaphase and the ER remained along the mitotic spindle. Flares of membrane began to protrude from the perispindle area. ER membrane was steadily lost from the area adjacent to the spindle pole over time (~20 minutes). Yellow trace line indicates plots for B, orange line for C. Scale bar is 5 μm. Time is in min:sec. (**B**) Fluorescence intensity trace plots of the longitudinal section of a nucleus. The plots are similar to wildtype through metaphase (see Fig. 1C). The later time points show a lack of intense ER at the poles, as seen in wildtype. (**C**) A plot of fluorescence intensity through a latitudinal section of the spindle highlights the increase in fluorescence around the spindle normally seen during mitosis. At the arrest, multiple peaks are seen where flares of ER expand from the perispindle region (arrows).

To address if mitotic ER dynamics are controlled by cyclin:Cdk1 machinery, we utilized a dominant-negative form of UbcH10 [36]. This dominant-negative mutant protein does not properly degrade cyclins A and B and arrests cells in metaphase. After injection of purified protein into embryos during mitosis of cycle 10, the syncytium proceeded through interphase and entered mitosis. As the syncytium progressed through mitosis, ER gathered around the spindle and formed cisternal clusters, while DNA condensed and aligned at the metaphase plate (Fig. 5A, S4 Movie). Mitosis stalled at this point, with chromosomes maintained as a very bright band at the spindle mid-zone and ER gathered brightly at the spindle periphery and poles. As quantified in Fig. 5B, the spindle remained ~10 μm long, but the accumulation of ER at the poles was lost over time. This could be due to the uncoupling of the centrosomes from the spindle during this arrest [37]. Notable during the arrest was the flaring of ER projections from the perispindle region (Fig. 5C, time point 20:00, arrows), indicating a loss of perispindle integrity as the arrest continued. The lack of ER dynamics progression by inhibition of the APC/C indicates that the morphological changes of the ER seen through mitosis are timed by cyclin:Cdk1 machinery.

### RNAi-inhibition of mitotic cyclins results in late mitotic defects on ER localization

**Fig 6:**
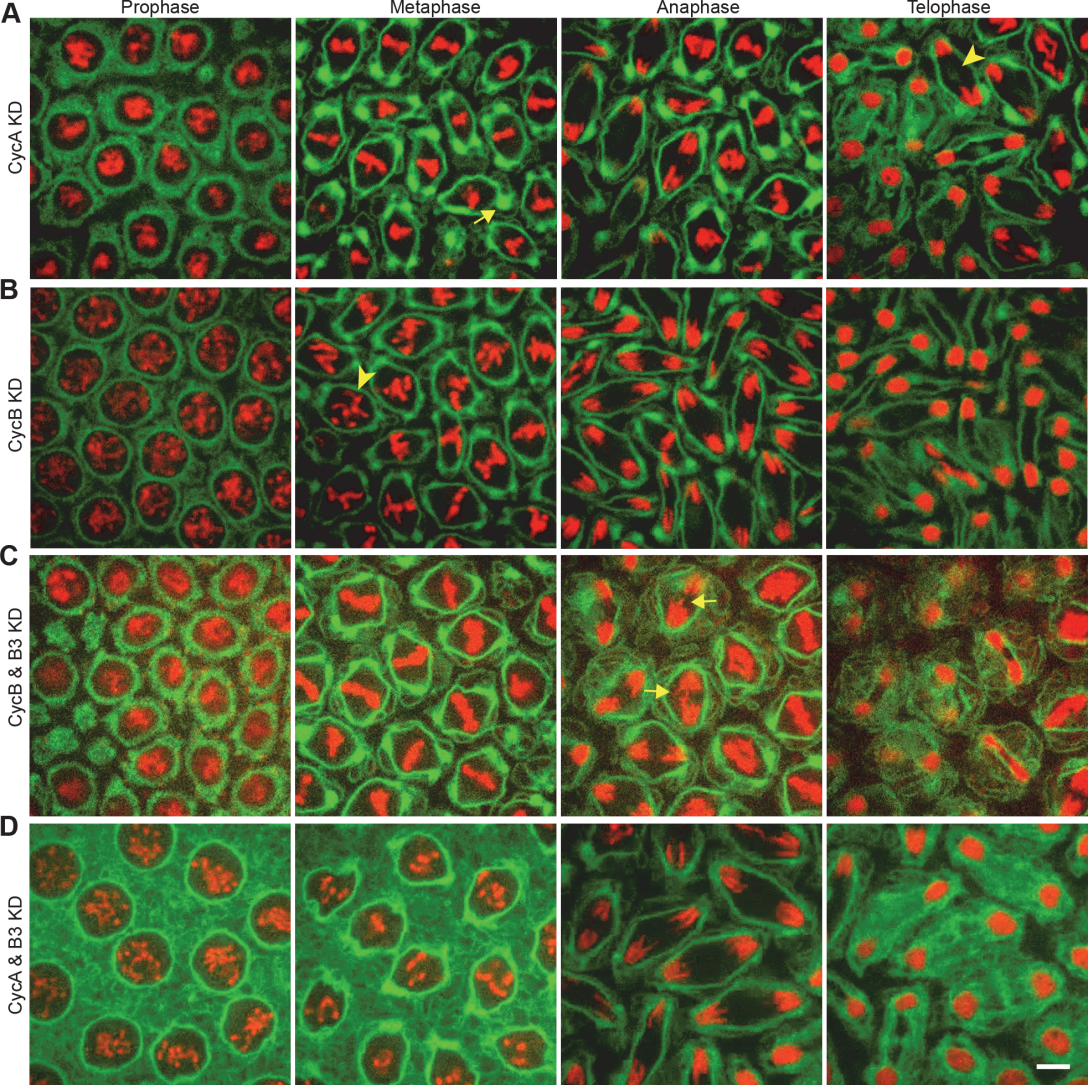
Mitotic ER Spatial Organization is Independent of Individual Cdk1 Activity. (**A**) Time-lapse confocal imaging of a Pdi-GFP (green) / H2-RFP (red) embryo injected with dsRNA corresponding to individual mitotic cyclins (cycle 7–9) and viewed during mitosis at cycle 12. When CycA was knocked-down, improper clustering of ER at the mitotic spindle was observed at metaphase (arrow), and nuclear envelope closure was uncoupled from telophase onset and ER invagination at the central spindle (arrowhead). (**B**) Knock-down of CycB caused irregular chromosome alignment at the metaphase plate (arrowhead). During telophase, the midbody of the ER was absent. (**C**) Pairwise knockdown of mitotic cyclins produced disparate ER phenotypes in mitosis. Knock-down of CycB and B3, leaving only CycA, did not arrest the cell cycle, but produced lagging chromosomes (arrows). (**D**) Double knockdown of CycA and CycB3 leaving only CycB during mitosis at cycle 11, saw uneven accumulation of ER around the spindle at prophase and metaphase. Scale bar is 10 μm.

We further investigated the role of the cyclin:Cdk1 pathway in controlling ER reorganization events by diminishing the amount of available cyclin protein via RNAi-mediated knockdown. We adopted a strategy developed by McCleland and colleagues [38] wherein expression of each of the three mitotic cyclins present in *Drosophila* was reduced via RNAi knockdown. dsRNAs targeting each of these cyclins were injected into Pdi-GFP / H2-RFP transgenic embryos (cycle 7–8) and imaged during cycle 10–13. Injection of a *LacI*-targeted dsRNA served as our control for these experiments (S3A Fig.). Knockdown of cyclin A (CycA) displayed defects in timing of mitosis, including a shortened prophase to metaphase transition as shown in previously published reports [39]. The ER displayed only minor defects in its localization at the perisindle region and poles during metaphase (Fig. 6A, arrow), however major defects were seen during telophase, including a mislocalization of ER membrane at the spindle midzone (Fig. 6A, arrowhead). Knockdown of cyclin B (CycB) showed defects in mitotic timing and coordination of nuclear division. Similar to CycA knockdown, there was a loss of ER localization at the spindle midzone at telophase (Fig. 6B). Unlike CycA knockdown, there were no noticeable effects on ER reorganization events early in mitosis.

Knock-down of individual cyclins (see S3 Fig. for CycB3 KD) did not inhibit overall cell cycle progression or ER dynamics. We then progressed to knocking down pairs of mitotic cyclins, to test whether the activity of a single cyclin was sufficient to drive mitotic events. Pairwise knockdown of CycB and B3, leaving only CycA, displayed defects including improper chromosome alignment and an increase in nuclei fusion events at telophase, similar to published reports [39]. Despite this, ER localization early in mitosis appeared normal (Fig. 6C), yet ER organization at the spindle midzone during anaphase and telophase was disrupted (Fig. 6C, arrows). Next, we performed pairwise knockdowns of CycA and CycB3, leaving only CycB. These embryos showed problems in nuclear spacing and a prolonged metaphase similar to published reports including an arrest in interphase of cycle 12 [39]. During mitosis of cycle 11, embryos displayed defects in accumulation of ER membrane early in prophase and a reduction of ER cisternae surrounding the spindle region at prometaphase and metaphase (Fig. 6D). Overall, we found that embryos deficient for two of the three mitotic cyclins displayed minor defects in proper ER localization early in mitosis anddisplayed a mislocalization of ER membrane at the spindle midzone during telophase.

### Cdk1 activity is necessary for changes in ER localization to the nuclear envelope and towards the poles

**Fig 7:**
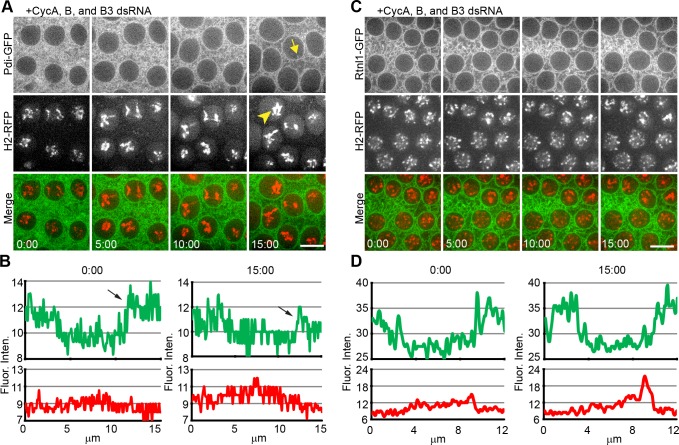
Mitotic Cyc:CDK1 Activity is Necessary for Mitotic ER Dynamics (**A**) Cycle 11 transgenic embryo expressing Pdi-GFP / H2-RFP following simultaneous dsRNA-mediated knockdown of Cyclins A,B, and B3. When all three mitotic cyclins were knocked-down, there was a general arrest of the embryo prior to entry into mitosis and a block in ER spatial reorganization events. ER tubules persisted between adjacent nuclei. Chromosomes incompletely condensed (arrowhead) and the ER occasionally invaded the nuclear space (arrow). (**B**) Quantification of the induced arrest from injection of dsRNA directed at all three mitotic cylins shown in A. Intensities of Pdi-GFP and H2-RFP fluorescence are represented by green and red, respectively. (**C**) Arrest of ER membrane dynamics was further confirmed by examination of the ER shaping protein, Rtnl1. Injection of dsRNA directed at all three mitotic cyclins into a Rtnl1-GFP / H2-RFP embryo produced an arrest prior to mitotic entry, indicating that Rtnl1 is able to change localization independent of mitotic cyclin/CDK activity. (**D**) Quantification of arrest seen in C with Rtnl1-GFP in green and H2-RFP in red. Scale bars are 10 μm. Time is in min:sec.

To examine the requirement of Cdk1 activity on mitotic ER reorganization, we injected Pdi-GFP / H2-RFP embryos with dsRNA targeting all three mitotic cyclins (cycle 7–8) and again imaged at a later stage over time-lapse. Simultaneous RNAi-mediated knockdown of all three mitotic cyclins caused a cell cycle arrest of the embryo at interphase of cycle 11 (3/3 embryos) (Fig. 7A, S5 Movie). During this arrested state, the chromatin displayed a hypercondensed phenotype (arrowhead) and freely diffused around the nucleus. This defect was reported in McCleland [38] and was shown, by lack of phospho-specific H3 staining, not to be mitotically condensed chromosomes. In this arrested state, the ER did not exhibit any gathering at the perispindle or poles normally seen during mitosis (Fig. 7A). This was also shown in quantitation of the fluorescence intensity of the ER (green), which showed a wide peak spreading between 10 μm and 15 μm, indicating a lack of accumulation at the poles (Fig. 7B, arrows). The fluorescence intensity remained relatively flat with no peaks of intensity normally corresponding to changes in localization along the nuclear envelope or at the poles. The embryo maintained this arrest for the duration of the time of observation (>30 min). During this time, the nuclei continued to swell to a very large size and tiny invaginations of the membrane occasionally invaded the nuclear space (arrow).

To further investigate the formation of ER at the spindle poles with knockdown of the mitotic cyclins, we created a transgenic line co-expressing Rtnl1-GFP and the DNA marker, H2-RFP. Rtnl1-GFP / H2-RFP embryos were injected with dsRNAs directed towards all three mitotic cyclins. With diminished mitotic cyclin activity, Rtnl1-GFP shows a similar arrest of the ER (5/5 embryos) (Fig. 7C). These data confirm that the mitotic ER reorganization events are dependent upon Cdk1 activity.

### Cyclin A activity is sufficient to initiate Mitotic ER reorganization Events

**Fig 8:**
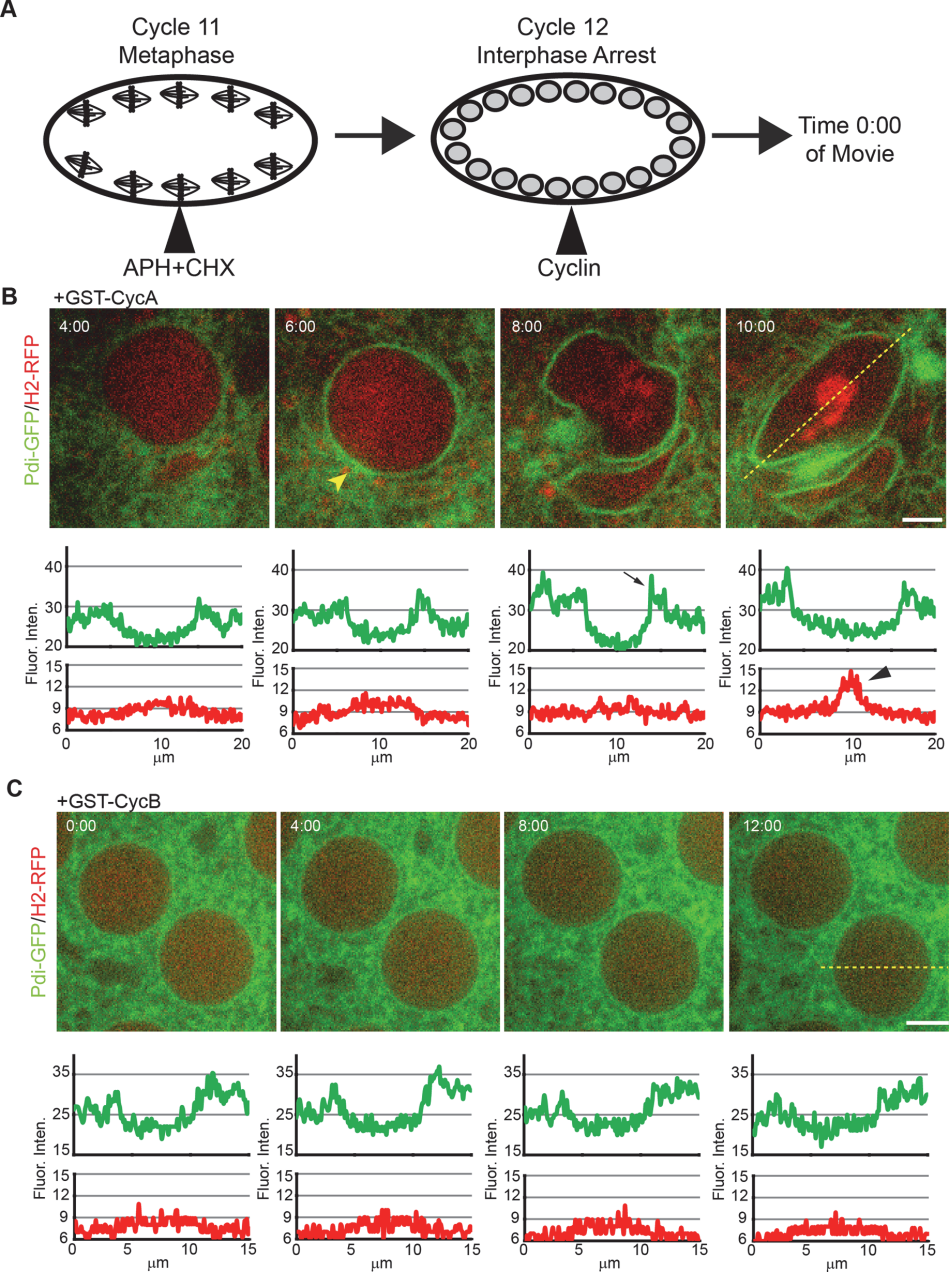
Cyclin A is sufficient to drive mitotic ER reorganization events. (**A**) Schematic of injection strategy and imaging of *Drosophila* embryo experiment. Pdi-GFP / H2-RFP transgenic embryos were injected with a mixture of APH and CHX, inducing a cycle 11 interphase arrest. Following this arrest, embryos were injected with an affinity-purified recombinant form of cyclin and observed for changes in ER localization. (**B**) After injection of GST-CycA, ER (green) gathered near the spindle (yellow arrowhead). Pdi-GFP intensity increases much like WT embryos (arrow). Chromosomes (red) eventually condensed and aligned at the metaphase plate (black arrowhead). The spindle region extended into a fusiform structure, but did not progress beyond this point. There was a lack of ER gathering at spindle poles, as well. (**C**) Following injection of GST-CycB, embryos remained in an interphase-like state without rearrangement of ER (green) or chromosome (red) condensation. Scale bars are 5 μm. Time is in min:sec.

We next wanted to determine if either CycA:Cdk1 or CycB:Cdk1 activity was sufficient to drive mitotic ER dynamics. We employed the strategy of arresting Pdi-GFP / H2-RFP expressing embryos in S-phase with a combination of the cell cycle inhibitors, APH and CHX. After the arrest was established (~10 minutes), a purified recombinant protein, either GST (control, S5A Fig.) or GST-cyclin (A or B), was injected into the embryo and imaged over time-lapse (Fig. 8A, S4 Fig.). In 80% (8/10) of CycA-injected embryos, the distal ER displayed a similar morphology to wild-type embryos beginning mitosis. Half of injected embryos (5/10) had the ER gather at the perispindle and poles of the spindle (Fig. 8B, arrow). Chromosomes condensed in 7 out of 10 embryos, consistent with a role in nuclear events [40]. Six of the embryos eventually progressed into metaphase where the ER took on a fusiform shape and chromosomes aligned at the metaphase plate, indicating the assembly of a spindle (Fig. 8B arrowhead, S6 Movie). None of the embryos injected advanced beyond metaphase.

Injection of GST-CycB following APH and CHX arrest did not show any effects on ER movement and dynamics and the ER remained in an interphase-like state (7/7 embryos) (Fig. 8C). We tested the same batch of GST-CycB protein injected into Pdi-GFP / H2-RFP embryos arrested only with CHX. Our GST-CycB possessed the ability to override a CHX-induced arrest and drive the embryo into a mitotic state similar to previously published reports [40,41], indicating the purified GST-CycB is active (S5B Fig.). Our results showed that CycA possesses the ability to initiate ER reorganization events during mitosis, while micro-injection of CycB did not demonstrate any changes to ER localization. These results indicate that CycA, usually found in the nucleus early in mitosis, is the regulatory cyclin responsible for the dramatic changes of the mitotic ER. We further suggest that this reliance on CycA provides a possible mechanism of timing of ER reorganization during prometaphase by the release of CycA from the nucleus into the cytoplasm at NEB.

## Discussion

As our understanding of cell cycle progression has advanced in recent years, an outstanding question yet to be fully addressed is the coordination of the cytoplasmic and nuclear events during mitosis. Here we focus on the dramatic structural transformation and regulation of the ER during mitosis in the early *Drosophila* embryo. The phosphorylation and subsequent degradation of cyclin:Cdk1 substrates controls the timing of many events during mitosis including phase progression [42], yet some mitotic events occur independently of this process [3]. For instance, chromosome condensation proceeds in the presence of a Cdk inhibitor, roscovitine [43]; likewise centriole replication and division largely depend on Polo kinase and Polo-like kinases (Plks) and not Cdk1 [44,45]. Seeing as most, but not all, canonical mitotic events occur through cyclin:Cdk1-mediated events, we were curious as to whether ER mitotic dynamics could be traced to particular cyclin activities if at all.

We present evidence that mitotic ER spatial reorganization seen during prometaphase is controlled by CycA:Cdk1 activity. Conversely, injection of a recombinant-CycB was not sufficient to drive the changes in ER localization seen during mitosis. A prior study involving the three mitotic cyclins (A, B, and B3) found in *Drosophila* indicated that each mitotic cyclin:Cdk1 pair targets and controls specific temporal events during mitosis [46]. In addition, dsRNA inhibition of all three mitotic cyclins blocked entry into mitosis, but did not disrupt localization of the ER shaping protein Reticulon-like 1 to the ER distal from the nucleus. Taken together this suggests that ER spatial reorganization is a cytoplasmic target of CycA activity and that timing of nuclear and cytoplasmic events during mitosis may also rely on the release of CycA from the nucleus during nuclear envelope breakdown during prometaphase as a mechanism of coordination.

### Cyclin A controls timing of ER reorganization events

The control and timing of critical events necessary to complete cell division has largely been viewed as the role of the mitotic cyclin:Cdk1 complexes [47,48]. The prevailing view is that CycB:Cdk1 controls the cytoplasmic events, while CycA:Cdk1 is responsible for nuclear events. This is largely due to their localizations immediately prior to mitosis where CycB shuttles between the nucleus and cytoplasm, while CycA is primarily inside the nucleus [2,40,49]. In addition, cytoplasmic CycB has been shown to localize with the mitotic spindle poles and Golgi apparatus suggesting a role in its function and activity during mitosis [40,50]. In contrast, CycA resides in the nucleus and has been implicated in early mitotic nuclear events until NEB where it is released into the cytoplasm in prometaphase and targeted for destruction by the anaphase-promoting complex (APC/C). Much of the studies surrounding the mitotic cyclins have focused on their regulation and destruction during mitosis and control of nuclear mitotic events [4,51,52] but an unanswered question is how or if these cyclins regulate discrete steps between the nucleus and the cytoplasm.

Our findings that CycA is sufficient to drive mitotic ER reorganization events in the early *Drosophila* embryo provides insight into this outstanding question and suggest that CycA can target and control cytoplasmic events. In addition, a possible mechanism of coordination and timing between the nuclear pool of CycA and timing of cytoplasmic changes driven by CycA may rely on the physical barrier of the nuclear envelope. Shuttling of CycB into the nucleus is linked to disassembly of the nuclear lamina and promotes NEB [6,53]. Interestingly, a recent study found that knockdown of the mammalian CycA2 delayed NEB and suggests a role for CycA in the timing of NEB [12]. After NEB, CycA is released into the cytoplasm to reorganize the ER membrane towards the spindle poles before its destruction. This idea is bolstered by the fact that the ER is congruent with the nuclear envelope and once the nuclear envelope is disassembled, the ER needs to transition to the mitotic spindle to remain in close proximity to the condensed chromosomes for reformation of the nuclear envelope and pore complexes at the end of mitosis. In support of a role for CycA involvement in ER dynamics is the finding of a population of CycA that localizes to the ER and the presence of a ER-associated CycA regulatory protein, SCAPER, in human cells that has been linked to cell cycle progression [54].

Surprisingly, our knockdown of CycA displayed only minor effects on ER spatial reorganization. One possibility is that this may be due to CycB3’s ability to substitute for CycA early in mitosis. In support of this is our result of the pairwise knockdown of CycA and CycB3 leaving only CycB displayed the most significant defects on ER localization during mitosis (Fig. 6D). In addition, expression of CycB3 overlaps with CycA early in mitosis indicating a possible partial redundant role in cyclin:Cdk control of ER dynamics [39,55]. Future experiments and the identification of ER-specific cyclin:CDK targets will help shed light a possible mechanism of ER control.

### Mechanism of ER spatial reorganization events during mitosis

Previous studies have suggested that the core cell cycle machinery may regulate the activity of the ER, including a halt in secretion during mitosis [9,10,17]. These studies focused on addition of cycB1Δ90 into an *in vitro* egg extract system or inhibition of general mitotic Cdk activity and showed a coupling of ER dynamics with the cell cycle. The targets of this cyclin:Cdk1 activity that drive ER dynamics are currently unknown. Possible candidates are the ER membrane shaping proteins: Reticulons, DP1/Yop1p, Atlastin and the recently described Lunapark [30,56,57]. Of particular interest is Atlastin, a member of the dynamin family of GTPases, because of its homotypic fusion activity, which makes it an excellent candidate for control of ER transformation. This is in light of recent evidence demonstrating that the yeast ortholog of Atlastin, Sey1p, is involved in regulating the interaction between reticulon (Rtn1p) and lunapark (Lnp1p) at ER junctions [57]. Another possibility is the ER associated microtubule-severing ATPase Spastin [58]. The association between the ER and the microtubule network has been well documented [23,59–61]. Additionally, Cdk1 has been implicated in regulation of microtubule dynamics during mitosis [40]. Spastin has been shown to sever and disassemble microtubules and has been linked to a group of neurodegerative disorders [58,62]. In addition, we have found that the aforementioned proteins all possess Cdk phosphorylation consensus sites (S6 Fig.). Future studies surrounding the regulation of these proteins at mitosis should lead to a molecular model of ER dynamics.

Work highlighted in this study of ER dynamics suggests that the control of cytoplasmic changes during mitosis relies on the spatial localization of the cyclins between the nucleus and the cytoplasm. Changes in the localization of these regulatory elements between the nucleus and cytoplasm leads to the sequential series of events that take place early in mitosis and the breakdown of the nuclear envelope may be the key event linking the nuclear changes to cytoplasmic changes.

## Materials and Methods

### Fly Stocks and Genetics

His2Av-RFP flies were a gift from Patrick O’Farrell (UCSF, San Francisco, CA). Pdi-GFP (#6839), UAS-RFP-KDEL (#30909), UAS-GFP-Lamin (#7376), UAS-mCherry-Tubulin (#25773), and a maternal triple driver (#31777) fly strains were obtained from the Bloomington Stock center (Bloomington, IN). Rtnl1-GFP[G00199] stock was obtained from the Flytrap database. Both the Pdi-GFP and Rtnl1-GFP lines were generated using protein-trap methodology (Morin *et al*., 2001). Homozygotes of Rtnl1[G00199]; His2Av-RFP or UAS-GFP-Lamin; UAS-RFP-KDEL or Rtnl1-GFP; UAS-mCherry-Tubulin were created by crossing individual fluorescent lines to balancer stocks and then mating the F1 progeny. UAS-GFP-Lamin; UAS-RFP-KDEL or Rtnl1-GFP; UAS-mCherry-Tubulin flies were mated to the triple driver strain and embryos from the F1 progeny were used for microscopy.

### Embryo Collection and microinjection

Embryos were collected on grape-juice agar plates, aged on collection plates, and dechorionated by hand. Dechorionated embryos were briefly desiccated and microinjected as previously described [41,63].

Needle concentrations for injected solutions were as follows: Rhodamine-labeled tubulin (Cytoskeleton Inc.) at 2 mg/mL, UbcH10-DN at 20 mg/mL [64], GST at 19 mg/mL, GST-CycA at 1 mg/mL, GST-CycB at 3.8 mg/mL, dsRNA at 500–1000 ng/μL [65], Cycloheximide (CHX) at 1 mg/mL, and Aphidicolin at 100 μg/mL. The following buffer was used to solubilize all proteins and small molecules prior to injection: 5 mM KCl, 0.1 mM NaPO_4_, pH7.5 as previously described [66].

### Imaging live Embryos by Laser Confocal Microscopy

Confocal images of injected embryos were obtained on an inverted microscope (Zeiss Cell Observer, Carl Zeiss Microimaging, Inc.) using the 488 nm and 543 nm wavelengths from an Argon laser. Images were captured with a C-Apochromat 1.2 NA 100x objective (Carl Zeiss MicroImaging, Inc.) and analyzed with ImageJ (W. Rasband, National Institute of Health [NIH], Bethesda, MD) and Axiovision (Carl Zeiss MicroImaging, Inc).

### Production of dsRNA

Double-stranded RNA (dsRNA) was made using the T7 RNA polymerase as previously described [67]. Templates for T7 transcription were created using a 2-step PCR. Primer sequences for Cyclin A, Cyclin B, Cyclin B3 are found in McCleland and O’Farrell [65]. Primers for LacI were made using Primer 3 software (MIT, Cambridge, MA). The linker sequence 5′- TAATACGACTCACTATAG -3′ was added onto the 5′ end of each primer to facilitate the addition of the T7 promoter in the second PCR reaction. dsRNA was phenol-chloroform extracted, ethanol precipitated, and resuspended in DEPC-treated water as previously described [38].

### Processing and Quantitation of Fluorescent Images

Single-plane, 8-bit images were generated by our microscopy methods. The images presented in the figures were adjusted for max intensity using ImageJ software. Areas of interest were cropped from the file and increased in size to zoom in using Adobe Illustrator. The quantitation of the raw data was done by drawing a one-pixel wide line through a single nucleus extending along the region of interest. The raw intensity of each channel along this line was then obtained using ImageJ software. Plots were created in Excel with the y-axis adjusted to the relevant range of values in order to highlight changes in intensity.

### Embryo fixation and 3D reconstruction

Pdi-GFP / H2-RFP transgenic embryos were fixed as described by [33]. For 3D reconstruction of the ER membrane, endogenous GFP and RFP expression was imaged on a LSM710 scanning confocal microscope (Carl Zeiss MicroImaging, Inc.). Images were acquired using a 63x oil-immersion objective / 1.40 NA, at 2.5x zoom. All image processing took place within the Zeiss Zen Lite software. Scanning images were taken at 0.1 μm z-steps to a depth of 10 μm. Images were processed for 3D surface view analysis using Slidebook 6.0 digital imaging processing software (3i Intelligent imaging innovations, Inc.).

## Supporting Information

S1 Fig Nuclear Envelope Stays Intact During Arrests. Time-lapse movie of a GFP-Lamin / RFP-KDEL embryo injected with APH and CHX during mitosis in cycle 10. The embryo arrested in an interphase-like state during the next cycle. ER (red) did not rearrange and the nuclear envelope (green) remained intact throughout the arrest. Scale bar is 10 μm and time is in min:sec.
(TIF)

S2 Fig Control injection buffer does not affect cytoplasmic or nuclear timing during mitosis. Injection buffer consisting of dH_2_O and 5 mM KCl, 0.1 mM NaPO_4_, pH7.5 was injected prior to entry into mitosis in Pdi-GFP (green) / H2-RFP (red) transgenic embryos. Injection buffer does not disrupt cytoplasmic events such as ER reorganization to the poles and perispindle region during mitosis. In addition, in the presence of injection buffer, chromosome segregation proceeds normally. Scale bar is 10 μm and time is in min:sec.
(TIF)

S3 Fig Additional Cyclin dsRNA-mediated knock-downs. (**A**) Time-lapse confocal imaging of ER mitotic dynamics in cycle 11 Pdi-GFP / H2-RFP transgenic embryos following injection of a *LacI* dsRNA sequence as a control. There were no observable defects seen. (**B**) Knock-down of CycB3 disrupted the gathering of ER around the spindle, especially at the poles. (**C**) Pairwise knockdown of cyclins A and B produced similar phenotypes as a single knock-down of CycA. Additionally, chromosomes did not align at a metaphase plate before anaphase.
(TIF)

S4 Fig Purification of GST-tagged Cylins. Coomassie stained SDS-PAGE of purified recombinant GST-Cyclin A (left) and GST-Cyclin B (right) used in this study. Molecular weights of the ladder band closest to fragment size is listed on the left.
(TIF)

S5 Fig Only GST-CycA Affects ER Dynamics in an APH+CHX Arrest. (**A**) After a Pdi-GFP / H2-RFP-expressing embryo was arrested in cycle 11 with APH and CHX, purified GST was injected as a control for the recombinant GST-tagged cyclin A and B proteins. There was no change in ER structure or organization, indicating the arrest was maintained despite the presence of GST. (**B**) Embryo expressing Pdi-GFP / H2-RFP was arrested in cycle 11 using only CHX. Purified GST-Cyclin B was injected and alleviated the arrest. ER (green) became tabulated in the cytoplasm and gathered at the spindle region while chromosomes condensed. Chromosomes (red) did not align at a metaphase plate and the ER did not form a stable fusiform structure. Further advancement into mitosis was not observed in any movie.
(TIF)

S6 Fig Cdk1 consensus sequences in ER-associated proteins. Reticulon-like 1 (Rtnl1), Spastin, and Atlastin, are proteins associated with the ER and affect the shaping of the ER. Each has a Cdk1-targeted consensus sequence (blue) with a Serine or Threonine residue that is a candidate for phosphorylation (bold, underlined).
(TIF)

S1 Movie Mitotic ER Dynamics in WT Embryos. Embryos expressing Pdi-GFP (green) and H2-RFP (red) were imaged during cycles 10 and 11. Frames were taken every 10 seconds. This video corresponds to Fig. 1A-B. Playback at 14 fps.
(AVI)

S2 Movie 3D reconstruction of ER structural organization at metaphase. 3D reconstruction of ER structure in a Pdi-GFP / H2-RFP embryo in metaphase of cycle 11. Sideview of ER distribution at metaphase. 90° clockwise rotation at 20 fps.
(WMV)

S3 Movie Interphase-like arrest of ER Dynamics. Embryos expressing Pdi-GFP (green) and H2-RFP (red) were injected with the protein synthesis inhibitor, cycloheximide (CHX) and a DNA synthesis inhibitor, aphidicolin (APH) at metaphase of cycle 10 and observed through an arrest during cycle 11. Frames were taken every 10 seconds. This video corresponds to Fig. 3C. Playback at 14 fps.
(AVI)

S4 Movie ER remodeling events require APC/C activity. Pdi-GFP (green) / H2-RFP (red) transgenic embryos were injected just prior to entry into mitosis with the UBCH10-DN APC/C inhibitor. Images are viewed as the embryo enters cycle 11 mitosis. Early mitotic events and ER dynamics were normal until the UBCH10-DN induced metaphase arrest. Frames were taken every 10 seconds. This video corresponds to Fig. 4A. Playback at 14 fps.
(AVI)

S5 Movie Inhibition of all three mitotic cyclins maintains ER reorganization in an interphase state. Pdi-GFP (green) H2-RFP (red) were injected with double stranded RNAs targeting all three mitotic cyclins (CycA, CycB, and CycB3) at cycle 8 and imaged during cycle 11. Knockdown of all three mitotic cyclins blocked entry into mitosis and maintained the ER reorganization in an interphase-like state. Frames were taken every 10 seconds. This video corresponds to Fig. 6A. Playback at 10 fps.
(MOV)

S6 Movie Cyclin A Can Drive APH+CHX Arrested Embryos into Mitosis. At the start of the movie, Pdi-GFP (green) and H2-RFP (red) expressing embryos were arrested with APH and CHX in cycle 11. At the break at 17:32 and 18:22 (frames 22 and 23), GST-CycA was then injected into the embryo. ER is seen gathering around the perispindle and poles while chromosomes condense and align at the metaphase plate. Frames were taken every 10 seconds. This video corresponds to Fig. 7B. Playback at 14 fps.
(AVI)
