# Supplementary figures and images for: Spatial Reorganization of the Endoplasmic Reticulum during Mitosis Relies on Mitotic Kinase Cyclin A in the Early Drosophila Embryo
Source: PLoS One. 2015 Feb 17;10(2):e0117859. doi: 10.1371/journal.pone.0117859 (PMC4331435; doi:10.1371/journal.pone.0117859)

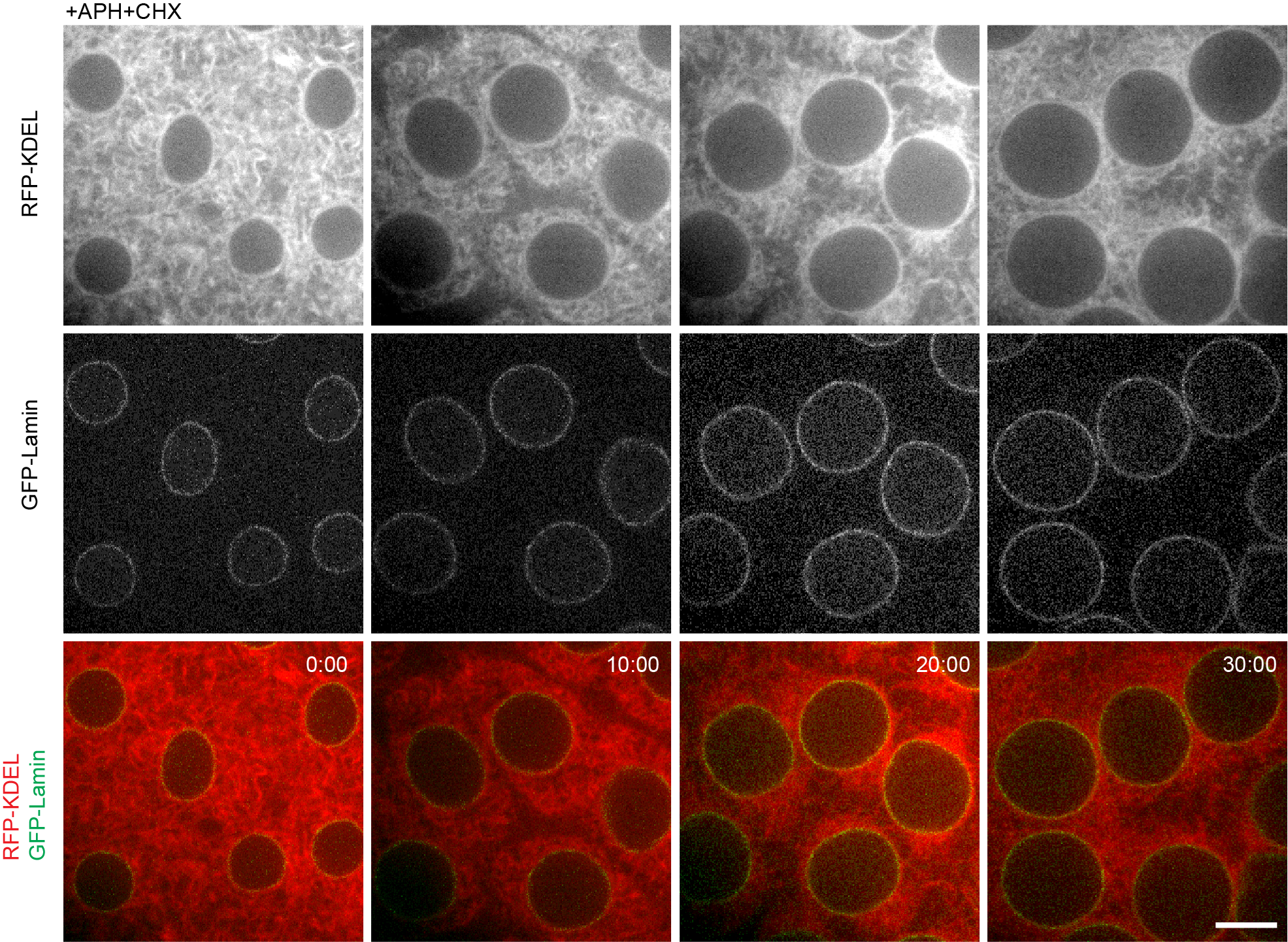

Supplement: S1 Fig — Time-lapse movie of a GFP-Lamin / RFP-KDEL embryo injected with APH and CHX during mitosis in cycle 10. The embryo arrested in an interphase-like state during the next cycle. ER (red) did not rearrange and the nuclear envelope (green) remained intact throughout the arrest. Scale bar is 10 μm and time is in min:sec. (TIF) [file pone.0117859.s001.tif]

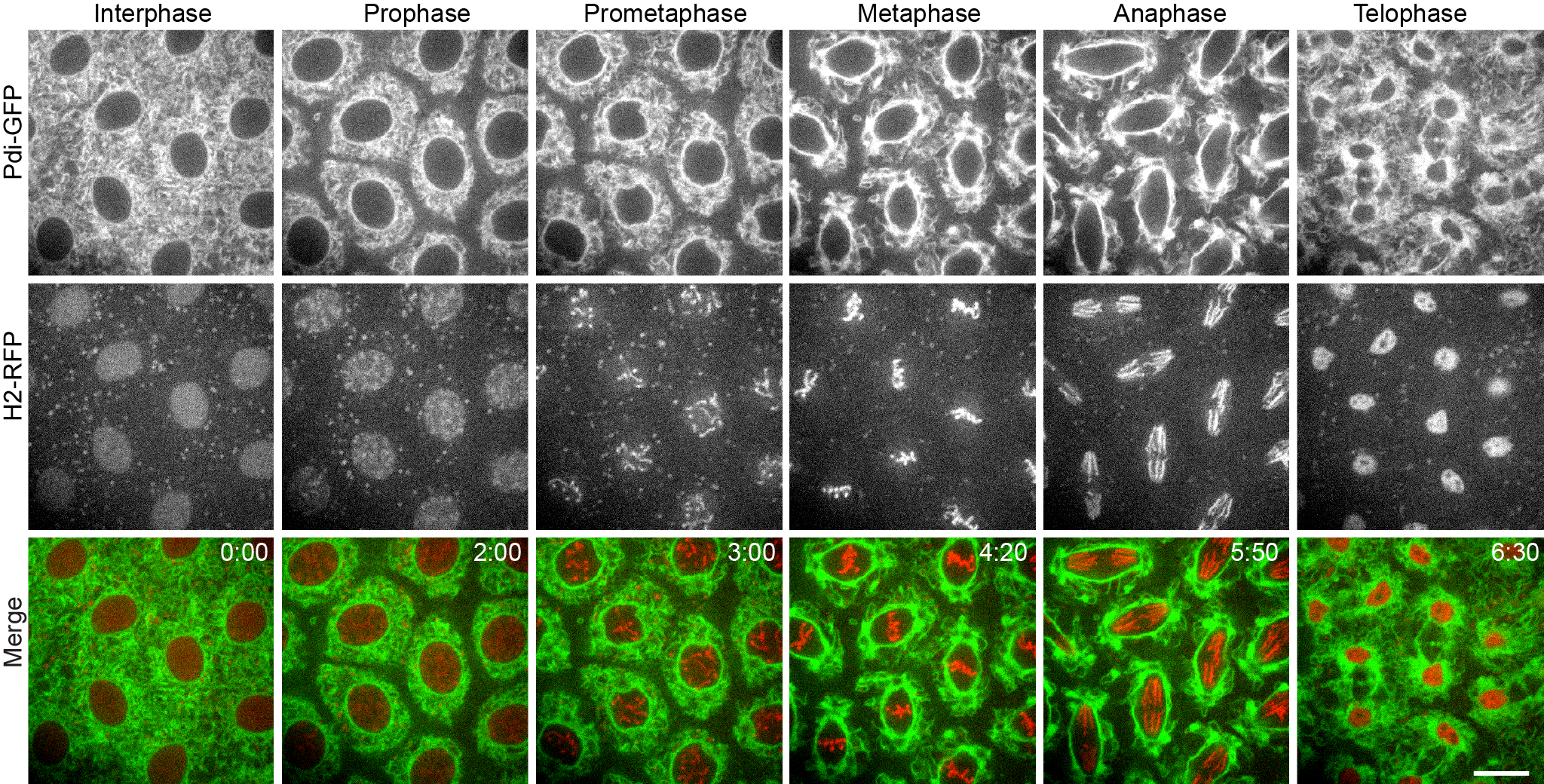

Supplement: S2 Fig — Injection buffer consisting of dH2O and 5 mM KCl, 0.1 mM NaPO4, pH7.5 was injected prior to entry into mitosis in Pdi-GFP (green) / H2-RFP (red) transgenic embryos. Injection buffer does not disrupt cytoplasmic events such as ER reorganization to the poles and perispindle region during mitosis. In addition, in the presence of injection buffer, chromosome segregation proceeds normally. Scale bar is 10 μm and time is in min:sec. (TIF) [file pone.0117859.s002.tif]

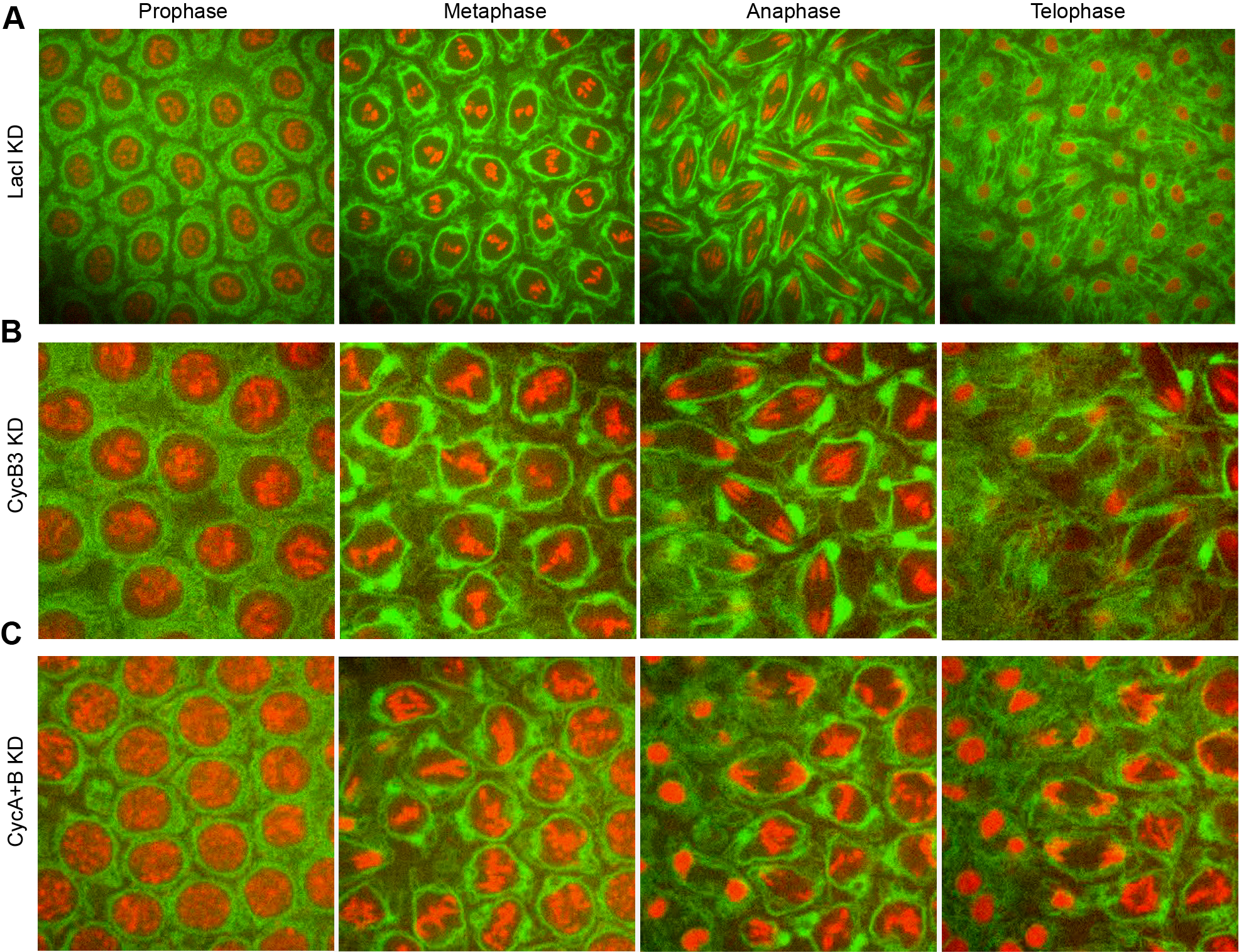

Supplement: S3 Fig — (A) Time-lapse confocal imaging of ER mitotic dynamics in cycle 11 Pdi-GFP / H2-RFP transgenic embryos following injection of a LacI dsRNA sequence as a control. There were no observable defects seen. (B) Knock-down of CycB3 disrupted the gathering of ER around the spindle, especially at the poles. (C) Pairwise knockdown of cyclins A and B produced similar phenotypes as a single knock-down of CycA. Additionally, chromosomes did not align at a metaphase plate before anaphase. (TIF) [file pone.0117859.s003.tif]

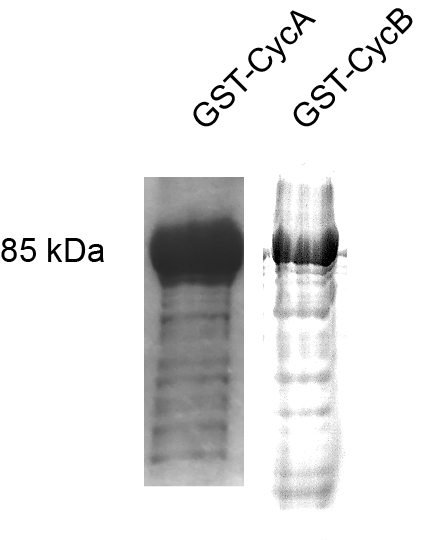

Supplement: S4 Fig — Coomassie stained SDS-PAGE of purified recombinant GST-Cyclin A (left) and GST-Cyclin B (right) used in this study. Molecular weights of the ladder band closest to fragment size is listed on the left. (TIF) [file pone.0117859.s004.tif]

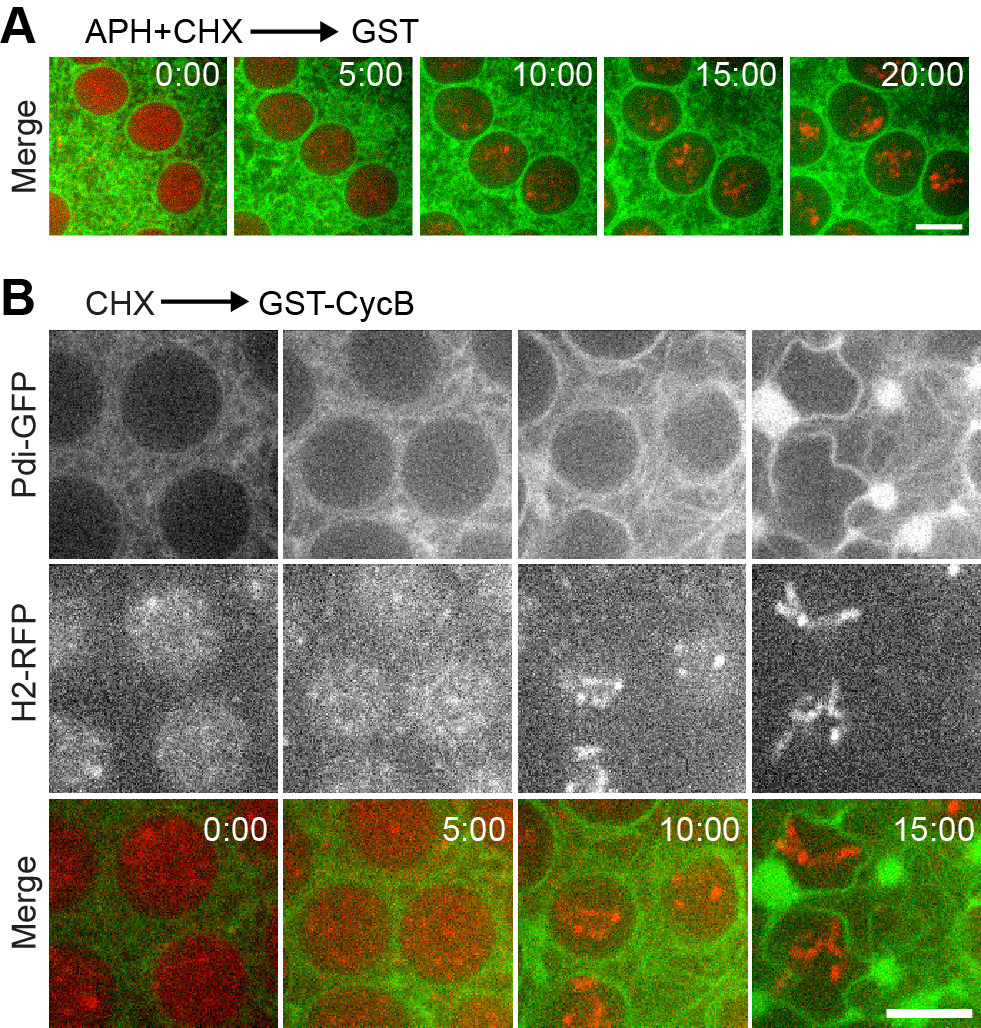

Supplement: S5 Fig — (A) After a Pdi-GFP / H2-RFP-expressing embryo was arrested in cycle 11 with APH and CHX, purified GST was injected as a control for the recombinant GST-tagged cyclin A and B proteins. There was no change in ER structure or organization, indicating the arrest was maintained despite the presence of GST. (B) Embryo expressing Pdi-GFP / H2-RFP was arrested in cycle 11 using only CHX. Purified GST-Cyclin B was injected and alleviated the arrest. ER (green) became tabulated in the cytoplasm and gathered at the spindle region while chromosomes condensed. Chromosomes (red) did not align at a metaphase plate and the ER did not form a stable fusiform structure. Further advancement into mitosis was not observed in any movie. (TIF) [file pone.0117859.s005.tif]

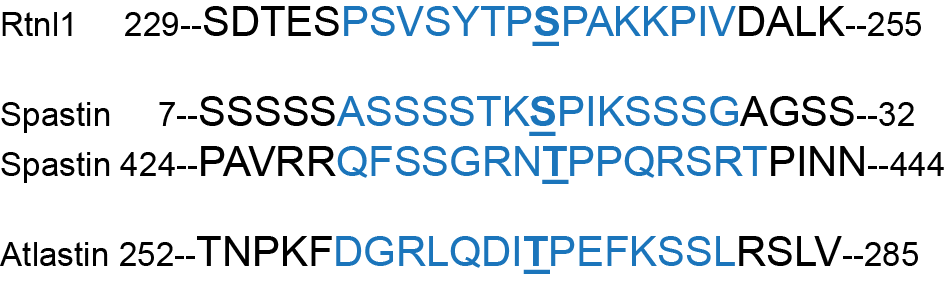

Supplement: S6 Fig — Reticulon-like 1 (Rtnl1), Spastin, and Atlastin, are proteins associated with the ER and affect the shaping of the ER. Each has a Cdk1-targeted consensus sequence (blue) with a Serine or Threonine residue that is a candidate for phosphorylation (bold, underlined). (TIF) [file pone.0117859.s006.tif]
